# Atypical cyclins: the extended family portrait

**DOI:** 10.1007/s00018-019-03262-7

**Published:** 2019-08-16

**Authors:** Eva Quandt, Mariana P. C. Ribeiro, Josep Clotet

**Affiliations:** Faculty of Medicine and Health Sciences, Universitat Internacional de Catalunya, Josep Trueta, s/n, Sant Cugat del Vallès, 08195 Barcelona, Spain

**Keywords:** Phosphorylation, Cell cycle, Cyclin family, Canonical, Transcriptional

## Abstract

Regulation of cell division is orchestrated by cyclins, which bind and activate their catalytic workmates, the cyclin-dependent kinases (CDKs). Cyclins have been traditionally defined by an oscillating (cyclic) pattern of expression and by the presence of a characteristic “cyclin box” that determines binding to the CDKs. Noteworthy, the Human Genome Sequence Project unveiled the existence of several other proteins containing the “cyclin box” domain. These potential “cyclins” have been named new, orphan or atypical, creating a conundrum in cyclins nomenclature. Moreover, although many years have passed after their discovery, the scarcity of information regarding these possible members of the family has hampered the establishment of criteria for systematization. Here, we discuss the criteria that define cyclins and we propose a classification and nomenclature update based on structural features, interactors, and phylogenetic information. The application of these criteria allows to systematically define, for the first time, the subfamily of atypical cyclins and enables the use of a common nomenclature for this extended family.

## Introduction

It is 35 years, since cyclins were identified as proteins that exhibited a cyclic pattern of expression throughout the cell cycle of sea urchin eggs [1]. This major breakthrough, which garnered Tim Hunt the 2001 Nobel Prize in Physiology or Medicine, together with Lee Hartwell and Paul Nurse, placed cyclins at the core of the cell cycle clock: cyclins expressed at different points form complexes with cyclin-dependent kinases (CDKs), a family of conserved serine/threonine kinases that phosphorylate different substrates throughout the cell cycle in an orderly manner.

Interestingly, although it is believed that early eukaryotes already exhibited complex mechanisms of cell cycle regulation, the number of both CDKs and cyclins significantly increased during the process of evolution (for an extensive review of the phylogenetic origin and degree of conservation of CDKs and cyclins across different species, see [2]). Whereas *Saccharomyces cerevisiae* possesses six CDKs, the human genome codifies at least 20 CDKs, each characterized by a catalytic core comprising the ATP-binding pocket, the PSTAIRE-like cyclin-binding domain and an activating T-loop motif [3]. While some CDKs play cell cycle-related functions (the CDK1 and CDK4 subfamilies), others have been implicated in transcription regulation (CDK7-13 and CDK19-20) (for a review of CDK nomenclature and classification, see [4]).

In humans, CDKs are partners of more than 30 cyclins, a remarkably diverse group of proteins that were initially named, because their protein levels fluctuate in a cyclical fashion during the cell cycle. Currently, they are solely defined by the presence of a cyclin box domain (CBD) [3]. In this review, we aim to introduce additional criteria for the definition of “cyclin proteins”.

The canonical CBD (PFAM 00134) is a sequence of approximately 100 amino acids arranged in five α-helices, and is responsible for binding and activation of CDKs [5–7]. Whereas all cyclins possess a CBD at the N-terminus half [8], some also exhibit an extra CBD (PFAM 02984 and PFAM 16899) which is thought to participate in protein folding [4, 9]. More recently, the C-terminal CBD of cyclin C was shown to mediate this cyclin’s binding to Drp1 GTPase, suggesting that the C-terminal CBD may be involved in CDK-independent functions [10]. In line with CDKs classification, cyclins have been traditionally divided in two groups, cell cycle (also frequently termed canonical) and transcriptional cyclins. The canonical cyclins (D, E, A, and B), which associate with cell cycle CDKs, were the first to be discovered and give the general name to the family [1]. The name canonical has been used by several authors working in different model organisms and pathologies [11–17] and reflects the fact that they share all the properties that originally defined cyclins: they are CDK activators that are periodically expressed and catabolized during different phases of the cell cycle [12]. They have also been named “cell cycle cyclins” [18], a designation that is better avoided as it excludes a “cell cycle” role for other cyclins (see below); moreover, it does not take into account that these cyclins regulate other aspects of the cell physiology beyond the cell cycle [19]. In contrast, the transcriptional cyclins (T, K, L, Q, C, and H) partner with transcriptional CDKs, and are mainly involved in the regulation of the RNA polymerase [20]; this designation has been broadly used by several groups working in the field [7, 11, 18, 21–25]. Nevertheless, although cyclins have been known by their ability to activate their partner CDKs, it is now recognized that either canonical or transcriptional cyclins carry out cellular functions that do not require the interaction with a CDK. For example, cyclin D1 may exert oncogenic actions through the stimulation of the transcriptional activity of estrogen receptors, independent of CDK4 [26], whereas cyclin C translocation to the cytoplasm promotes mitochondrial fission in yeast in response to stress [27].

However, over the last years, Genome Projects of different organisms unveiled the existence of several other proteins bearing a CBD [25], which remain poorly characterized. These novel members of the cyclin family have been named *new*, *orphan,* or *atypical* cyclins, reflecting different aspects of their distinctive features: the recentness of their discovery, the ignorance of their CDK partner, or some structural specificities, respectively. Moreover, proteins as diverse as the Transcription Factor IIB (TFIIB) or retinoblastoma (Rb) have an evolutionary distant CBD [5, 6] and, consequently, they are unlikely to work as CDK activators. To further increase the complexity, it is now recognized that some proteins lacking the CBD are also able to activate CDKs, including viral cyclins, specific CDK5 activators, and RINGO/Speedy proteins. These CDK activators, which were the subject of an excellent review by Nebreda [28], lack the CBD that characterizes cyclins and, therefore, are out of the scope of this work, as discussed later in the manuscript.

Therefore, the state of the art in the field supports the need to revise the criteria that have been used to classify cyclins, in line with previous efforts to create a systematic nomenclature for CDKs [29].

In this review, we perform a comprehensive analysis of the human cyclin family. The identification of structural similarities and particular interactor patterns enabled us to revise the existing classification and propose a new one that includes the novel members of the family.

## New cyclins: who is part of the family?

**Fig. 1:**
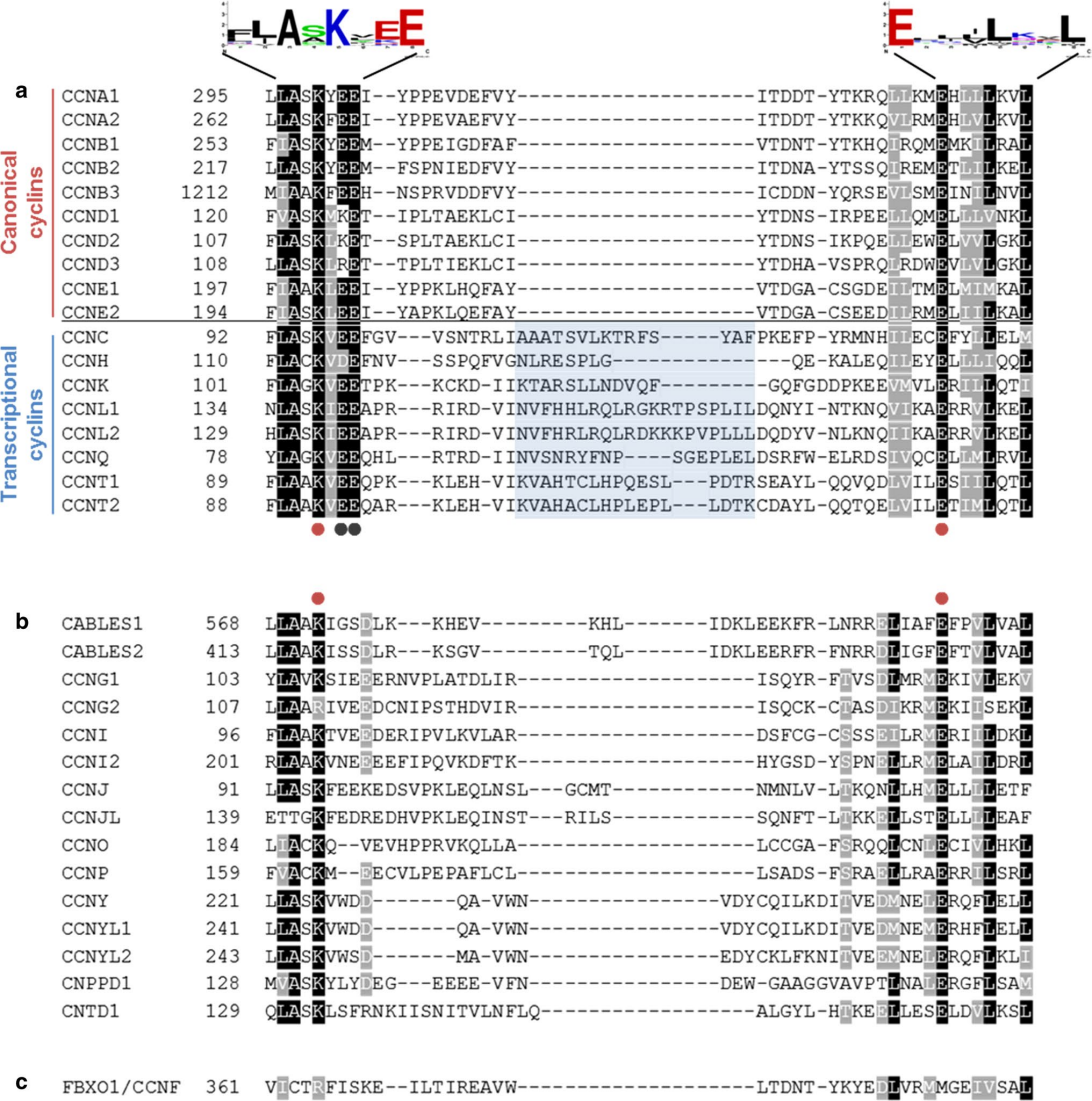
Conservation of critical residues for CDK binding. Multiple sequence alignment of the cyclins region critical for CDK binding [30] was conducted using *Jalview* software set to *Clustal Omega*. The resulting alignment (manually refined) was grouped into canonical and transcriptional cyclins (**a**) as they are considered *bona fide* cyclins, or potential cyclins (**b**) and exported to *ExPASy BoxShade* to highlight conservation (black boxes = conserved). **c** Cyclin F (CCNF)/F-box only protein 1 (FBXO1) sequence alignment. Logos for cyclin consensus sequences were identified using WebLogo [112]. Red dots indicate the Lys–Glu pair conserved among cyclins; gray dots indicate the Glu–Glu pair that is only present in canonical and transcriptional cyclins. Aminoacid sequences were retrieved from UniProt; positions of the N-terminal cyclin boxes used for the alignment were retrieved from Pfam, a database of curated protein families defined by multiple sequence alignments and hidden Markov model profiles [113]

There is now a significant body of evidence that supports that both canonical and transcriptional cyclins are *bona fide* members of the cyclin family, as they bear a canonical CBD and they are known to be part of established CDK-cyclin complexes. These two groups of cyclins show significant conservation within the CBD region near residues K257 and E286 of CCNB1 (indicated by red dots in Fig. 1a), which were described to be critical for CDK binding in the six CDK/cyclin complexes that have known crystallographic models [30]. Indeed, mutation of this lysine in the CBDs of cyclin D1 and cyclin Y impairs the interaction with their respective CDK partners [30, 31]. A close look at this region reveals that there are also other well-conserved residues, of which there is less experimental evidence (Fig. 1a), and that transcriptional cyclins have a characteristic insertion between these two residues that is absent in canonical cyclins (Fig. 1a); such structural particularity may help to explain the specificity of interaction with distinct CDK subfamilies.

The defining Lys–Glu pair is remarkably conserved among the novel members of the cyclin family (Fig. 1b). Therefore, we propose that besides having a CBD (PFAM 00134, PFAM08613), a protein should contain the characteristic Lys–Glu pair to be considered a cyclin. Cyclin F (CCNF) does not have this critical Lys; it neither possesses the defining Glu nor the other surrounding conserved residues (Fig. 1c). For this reason, we consider that CCNF, which has been included in the family of F-box proteins [32], should not be part of the cyclin family, as it is unlikely that it interacts with a CDK. In line with these observations, the designation of F-box only protein 1 (FBXO1) would be a more adequate name for this protein, and has already been used in the literature [14, 33–35].

The unique exception is CCNG2, which does not present the characteristic Lys, but has another positively charged polar amino acid (Arg) instead; still, it presents the defining Glu and a significant degree of conservation on surrounding residues, in contrast to CCNF (Fig. 1) and, therefore, we consider that it should remain part of the family.

All the other potential cyclins analyzed conserve the defining Lys–Glu, suggesting that they are capable of interacting with CDKs and should thus be included in the cyclin family and named accordingly. Noteworthy, the majority of the novel members of the family present some particularities in their sequences, such as the lack of the double glutamic acid (indicated by two gray dots in Fig. 1a) located in close proximity to the defining Lys of both canonical and transcriptional cyclins. Although it is not yet clear how this distinctive feature affects the interaction with a CDK, it is tempting to speculate that these cyclins might interact with different CDKs. Therefore, we will tentatively name all the new members of the family as atypical cyclins, a matter that is further discussed throughout the manuscript.

A no less important issue is the nomenclature of atypical cyclins. Whereas canonical cyclins were named by alphabetical order of appearance, the names of some atypical cyclins have a tortuous history, and some of these proteins even had several names at the same time, as demonstrated by the following examples. First, CCNL2 was previously known as CCNS, described as having a high homology to CCNL [36]; they are now considered isoforms. Second, the cyclin that was originally named CCNP by Murray and Marks [25] disappeared from the repositories after successive annotations; later, it was annotated again in the human genome but with a new name, CNTD2. The use of CCNP is also accepted by the HUGO Genome Nomenclature Committee (https://www.genenames.org/data/gene-symbol-report/#!/hgnc_id/HGNC:25805). Given that at the present moment, there are only five PubMed entries using this name, we propose that CCNP should again be considered as the first name option. Third, CCNQ has been named FAM58A and cyclin M, a designation that should be avoided given that it is still used to refer to metal transporters, which were initially named Cyclins M1-4. Finally, CCNY isoform 3 was formerly known as CCNX [37].

## Human cyclin interactors: who is my CDK partner?

**Fig. 2:**
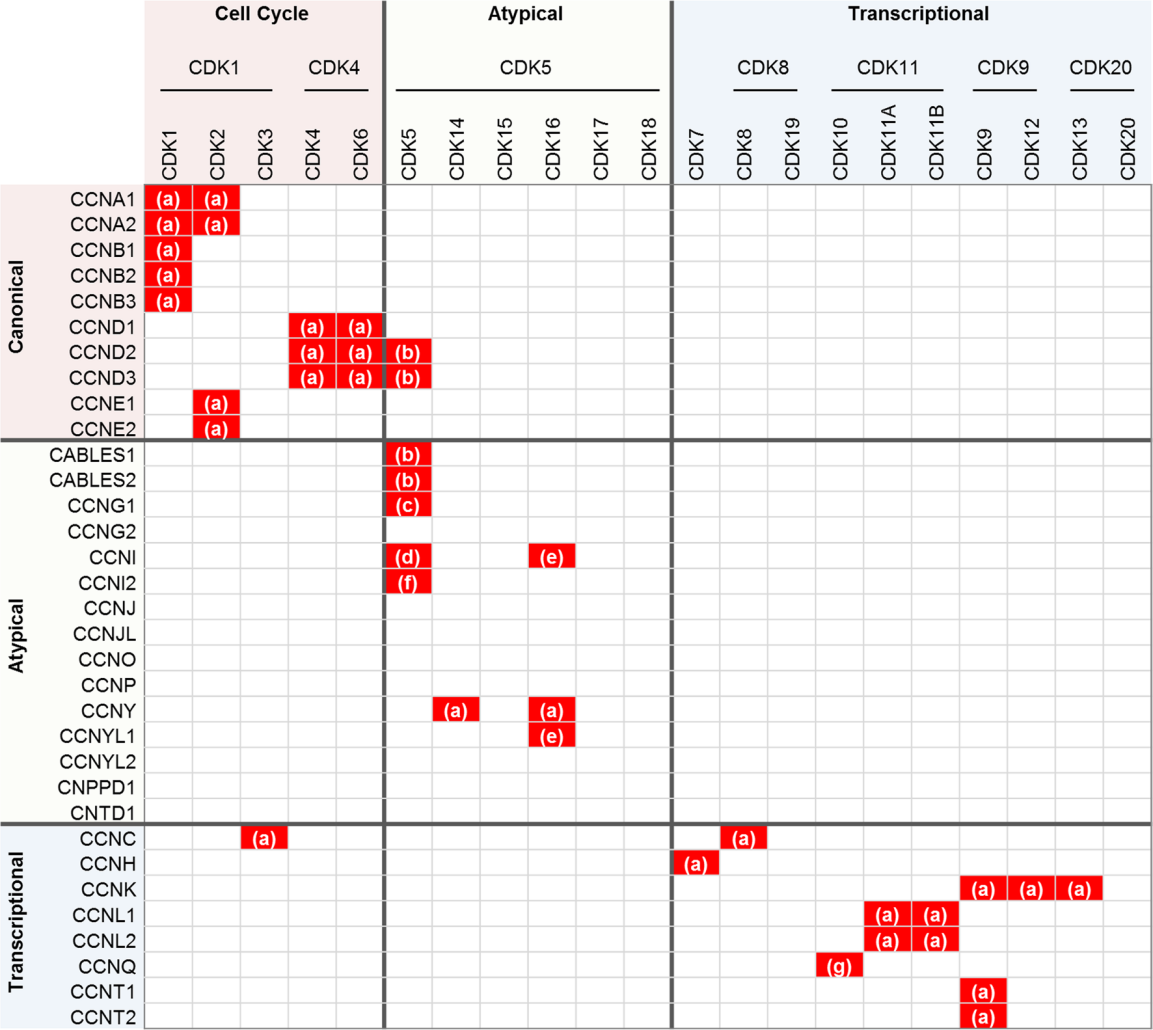
CDK–cyclin interactions. Cyclins were grouped into canonical, transcriptional, and atypical cyclins, whereas CDKs were grouped into cell cycle, transcriptional, and atypical CDKs as described by Malumbres, 2014 [4]. The table shows the reported CDK–cyclin interactions. For atypical cyclins, a systematic search in the STRING database was conducted [114]. Only the interactions supported by experimental data were selected and further revised for confirmation. **a** reviewed in [3]; **b** [115]; **c** [54]; **d** [66]; **e** [84]; **f** [70]; **g** [116]

Consistent with the distinctive features of the region that determines CDK binding (Fig. 1), canonical and transcriptional cyclins interact with different CDKs (Fig. 2). Canonical cyclins have interactors among the CDK1- and CDK4-related subfamilies, which is coherent with their role in cell cycle regulation, while transcriptional cyclins interact with transcriptional CDKs from subfamilies 7, 8, 11, 9, and 20, as expected from their functional role (Fig. 2).

In contrast to the previous ones, a significant number of atypical cyclins remain “orphan”, with no known partner CDK, a term that was used to refer to “orphan CDKs”. Interestingly, when an interactor for the atypical cyclin has been identified, it belongs to the CDK5-related subfamily (Fig. 2), a group that includes the so-called atypical CDKs: 5, 14, 15, 16, 17, and 18 [4]. This predilection for the CDK5 subfamily of CDKs suggests that the “atypicals” are likely to play roles that go beyond mere redundancy with the other subfamilies of cyclins, participating in specific cellular processes. Nevertheless, this hypothesis is limited by the absence of studies that systematically address the interactions between cyclins and CDKs, and it is possible that more complexes are yet to be identified.

## Structural identity of human cyclins

**Fig. 3:**
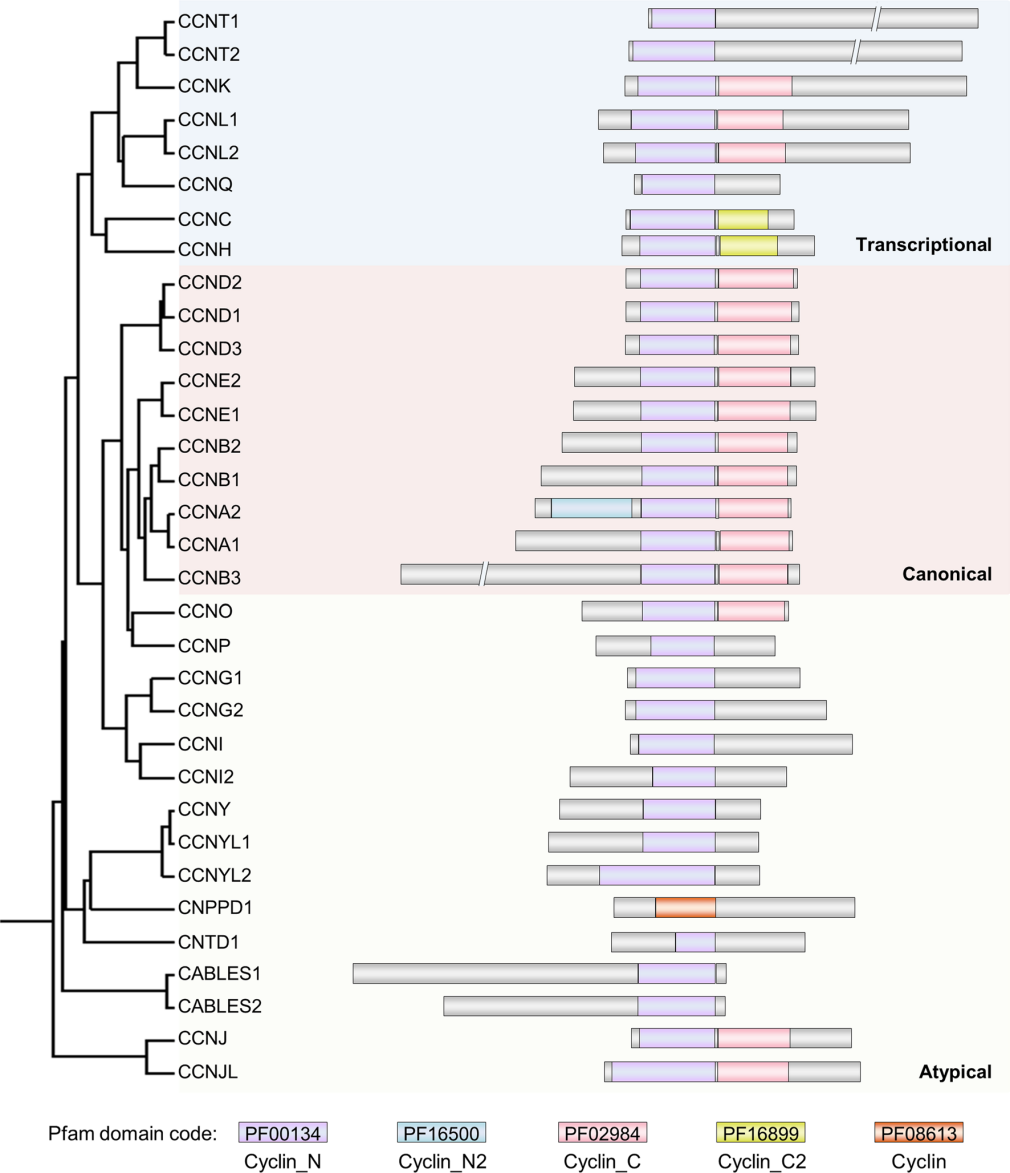
Human cyclin proteins. Sequence-based phylogenetic tree of human cyclins. The tree was generated by bootstrap analysis of the N-terminal cyclin box amino acid sequence using *Clustal Omega*. All the proteins containing either a Pfam domain PF00134 or PF08613 were included in the analysis. The length of the proteins, as well as the length of the domains, are on scale

The identity of the N-terminal CBD was selected to establish the relationships between family members, given that this is the most conserved region [8]. With this type of analysis (Fig. 3), the transcriptional cyclins emerge as a group with a clear identity in line with previous alignments [2, 11, 38]. Also as described, all the canonical cyclins present high homology in their CBD. Interestingly, some of the atypical cyclins (O, P, G, and I) have a CBD closely related to canonical cyclins, whereas the other atypical members seem more distant, suggesting that atypical cyclins might have different evolutionary origins.

Cyclins architecture is highly variable in terms of length, with some members presenting extensions on either the N- or the C-terminals of the CBDs (Fig. 3). Most remarkable is the fact that although closely related in terms of sequence identity of the N-terminal CBD, the majority of atypical cyclins present a single CBD, while all the members of the canonical subfamily have two.

At that point, we propose to establish the new subfamily of atypical cyclins whose members should fulfill at least two of the following criteria: they present a specific context of amino acids in the vicinity of the defining Lys–Glu pair (Fig. 1); they are still “orphans” or interact with atypical CDKs (Fig. 2); and they only have one CBD (Fig. 3).

## Atypical cyclins for novel functions?

After establishing the new subfamily of atypical cyclins, the analysis of their main functions may eventually shed some light on the need to have additional cyclins.

Early studies demonstrated that CABLES1 is located in chromosome 18q, which is commonly lost in colon cancer [39], suggesting that CABLES1 played an important role in this malignancy. Indeed, later studies demonstrated that loss of CABLES1 enhanced tumor progression in the Apc(Min/+) mouse model, which may be a consequence of increased activation of the Wnt/β-catenin pathway [40]. It was also shown that CABLES1 overexpression induced apoptosis and inhibited cell growth at least in part through p21 stabilization [41, 42], further consolidating that CABLES1 acts as a tumor suppressor. Moreover, CABLES1 has been shown to protect p63 from proteasomal degradation, modulating its function during genotoxic stress [43]. The role of CABLES1 in cancer was recently revised by Huang et al. [44]. Interestingly, CABLES1 has been mostly regarded as an adaptor protein and most studies have not addressed the relevance of its interaction with CDKs for its cellular functions.

In contrast to CABLES1, the role of CABLES2 has been much less investigated. However, an early report suggests that it may also act as a proapoptotic factor, via both p53-dependent and independent pathways [45]. Although it was reported that the CBD plays a role in CABLES2-induced apoptosis [45], it was not fully demonstrated that these effects are mediated through binding to CDK5, which was described as its main CDK partner [46].

CCNG1 transcription is activated by p53 [47, 48], which is consistent with its proposed role in apoptosis and growth inhibition [49, 50]. Likewise, CCNG1 decreased the proliferation of human endometrial cells [51] and increased sensitivity to radiation [52]. CCNG1 also works as a negative regulator of p53 through binding to PP2A and consequent Mdm2 dephosphorylation [53]. It was also reported that CCNG1 rendered lung cells more susceptible to DNA damage by upregulating cyclin B1 [52]. It was later proposed that CCNB1 transcription was upregulated by the CCNG1/CDK5 complex via phosphorylation of c-Myc on Ser-62 [54]. The evolving function of CCNG1 and its potential as therapeutic target in cancer have been the object of a recent review by Al-shihabi et al. [12].

CCNG2 was shown to inhibit cell cycle progression by binding to PP2A (through a region outside the CBD) and modulating centrosomal-associated activities [55, 56]. CCNG2 was also implicated in cell differentiation by promoting syncytiotrophoblast differentiation [57] and terminal differentiation at the site of blastocyst after implantation [58]. Moreover, CCNG2 inhibits cancer stem cell-like properties and suppresses the epithelial-to-mesenchymal transition by attenuating Wnt signaling [59, 60]. CCNG2 downregulation was also associated with chemoresistance [60]. Consistent with its tumor suppressive role, which has been associated with lower CDK2 protein levels, the expression of CCNG2 is downregulated in several human cancers, and correlates with a worse clinical prognosis [61–64].

CCNI was shown to regulate the survival of podocytes, which are essential for the integrity of kidney glomeruli [65]. The prosurvival function of the CCNI-CDK5 complex was shown to occur via MEK/ERK pathway activation and upregulation of antiapoptotic Bcl-2 and Bcl-XL [66]. CCNI has also been shown to increase the proliferation of Hela and lung cancer cell lines, although it is still unclear whether these effects are mediated through the interaction with a CDK [67, 68]. Accordingly, CCNI overexpression was associated with a worse prognosis in patients with lung adenocarcinoma [67] and it was proposed that CCNI mRNA in saliva could be a biomarker for lung cancer detection [69].

CCNI2 was recently shown to regulate cell proliferation, acting as a CDK5 activator, similarly to CCNI [70]. It was proposed that CCNI and CCNI2 compete for binding to CDK5, determining distinct subcellular localizations [70]. Considering that CDK5, in addition to its protective role in neuronal tissue [71], is implicated in the initiation and progression of neuroendocrine thyroid cancer [72], it would be interesting to explore the differential function of these complexes in the context of cancer and uncover potential strategies targeting CDK5-mediated oncogenic signaling.

It was reported that CCNO is expressed in the cytoplasm of multiciliated cells, critical for mucociliary clearance, acting downstream of multicilin [73]. Cells with impaired CCNO expression display a marked reduction in the number of multiple motile cilia, which is caused by altered generation of centrioles at deuterosomes [73, 74]. Therefore, it is not surprising that several studies have assigned CCNO a role in multiciliogenesis and mucociliary disorders [73–78] and infertility [79]. CCNO has also been shown to promote apoptosis in lymphoid cells [80].

Recent work from our group demonstrated that CCNP, also known as CNTD2, is overexpressed in samples from lung and colon cancer patients and promotes tumor cell proliferation and migration both in vitro and in vivo [67, 81]. Further studies are now required to provide insight on the mechanisms underlying the oncogenic actions of CCNP. Apart from these studies, it is only known that CCNP enhances viral replication [82] and correlates with the expression of Epstein–Barr virus genes [83].

CCNY was shown to be essential for spermatogenesis [84] and to play a role in cytoskeleton regulation either in complex with CDK16 [85] or CDK14, the latter involving activation of non-canonical Wnt signaling [86]. The ability of CCNY to activate Wnt signaling was also shown to be critical for maintenance of progenitor cell properties during cell division [87]. Recently, it was reported that the CDK16/CCNY complex increased the proliferation of several cancer cell lines through the phosphorylation of the protein regulator of cytokinesis 1 (PRC1) [85]. Despite this role in cell proliferation, CCNY is also expressed in neurons, where it is associated with several functions, including the regulation of synaptic plasticity [88, 89].

Like CCNY, CCNYL1 has been implicated in the maintenance of stem cell properties by activating Wnt signaling in mitosis [87]. CCNYL1, in complex with CDK16, was also implicated in spermatogenesis [90], a finding that may also reflect its ability to modulate Wnt signaling [91].

Regarding CNTD1, it was shown to be critical for meiotic crossover maturation by regulating the association between HEI10 and RNF212 and components of the crossover machinery [92].

Remarkably, the role of some atypical cyclins remains largely unexplored. This is the case of CNPPD1, which is mentioned as a gene candidate for schizophrenia susceptibility in the Japanese population [93]. More recently, CNPPD1 was proposed as a neoantigen in breast cancer lymph node metastasis [94]. Regarding CCNJ, it is known to be repressed by tumor suppressor microRNAs in breast, gastric, prostate, and bladder cancers [95–98]. At the present moment, there are no entries in PubMed that may shed some light on the roles of either CCNYL2 or CCNJL.

Whereas the functions of most atypical cyclins are still poorly characterized, many of them have also been implicated in key cellular processes that have been attributed to canonical cyclins [19], such as cell proliferation and differentiation, or response to DNA damage (Table 1). Although this review focuses on human proteins, we have confirmed that most of the cyclins are conserved among mammals (not shown). Moreover, some atypical cyclins, such CCNYs (named PCLs in yeast), are conserved in unicellular eukaryotes. Such high degree of conservation is consistent with an essential role [2]. Interestingly, some atypical cyclins appeared later in evolution (CCNG1/I, CCNJ, or CCNO), but so did some canonicals, such as CCNA [2], suggesting that these late-appearing atypical cyclins may perform specific functions rather than being mere accessories to the normal cell physiology.

**Table 1:** Main functions of atypical cyclins

Cyclin	Function	Main references
CABLES1	Modulation of response to genotoxic stress	[43]
	Regulation of cell proliferation and apoptosis	[40–42]
CABLES2	Apoptosis promotion	[45]
CCNG1	Participation in response to DNA damage	[117–119]
	Regulation of cell proliferation and apoptosis	[49, 50]
	Regulation of cell cycle progression	[55, 56]
CCNG2	Regulation of cell differentiation	[57–59]
	Regulation of DNA damage response	[120, 121]
CCNI	Protection of kidney podocytes from apoptosis	[65, 66]
	Regulation of cell cycle progression	[67, 68]
CCNI2	Regulation of cell cycle progression	[70]
CCNO	Apoptosis induction in lymphoid cells	[80]
	Regulation of deuterosome-mediated amplification of centrioles in multiciliated cells	[73, 74]
CCNP	Regulation of cancer cell proliferation	[67, 81]
	Modulation of viral replication	[82]
	Regulation of spermatogenesis	[84]
CCNY	Regulation of neuronal function	[88, 89, 122]
	Maintenance of stem/progenitor cell properties	[87]
	Regulation of cytoskeleton and cell proliferation	[85, 86]
CCNYL1	Regulation of spermatogenesis	[90, 91]
	Maintenance of stem/progenitor cell properties	[87]
CNTD1	Essential for meiotic crossover maturation	[92]

Further studies are now needed to fully characterize the roles of atypical cyclins in the physiological context. The recent observation that embryonic stem cells are able to proliferate in the absence of G1 cyclins [99], suggests that other (perhaps atypical) cyclins may be able to drive cell cycle progression in this cellular model. On the other hand, while additional cyclins are likely to be advantageous in a physiological context by providing additional regulatory flexibility, such diversity may actually be deleterious in a pathological context. For instance, the complexes of CCNY with either CDK14 or CDK16 have been shown to increase cell proliferation [85, 100, 101], suggesting that at least some atypical cyclins can use alternative CDKs to foster cell proliferation. Furthermore, atypical complexes may provide an escape route to the inhibitory actions of anticancer agents, in agreement with the observation that CCNG1 downregulation increased sensitivity to doxorubicin [102] or paclitaxel [103].

## Concluding remarks

The complexity of the cyclin family increased dramatically over the last 2 decades. With so many cyclins, it became unclear what defines cyclins and how new members can be integrated in this family.

Cyclins owe their name to their cyclic pattern of expression [1]; this defining trait soon failed to embrace the distinct expression pattern of transcriptional cyclins, the levels of which do not oscillate. On the other hand, cyclins have been defined by their biological roles in cell cycle or transcriptional regulation, which seems to be overly simplistic given that “cell cycle” cyclins also modulate transcription [19]. Therefore, our classification is based on the presence of a Lys–Glu pair that is known to be critical for interaction with a CDK (Fig. 1). It is important to highlight that our criteria for inclusion in the cyclin family were mostly based on structure, rather than function (ability to activate CDKs); this way, proteins that lack the characteristic N-terminal CBD were excluded from this analysis, even though they may work as CDK activators. For example, p35, a known activator of CDK5 lacks significant sequence homology with cyclins [28]. Another example would be the RINGO/Speedy proteins that, in spite of lacking a CBD, are also able to activate CDK1 and CDK2 [28, 104, 105]. The fact that CDK activation by RINGO/Speedy proteins is CAK1 independent [28, 106, 107], suggests that their activation mechanism is distinct from the one used by canonical cyclins. This observation, together with their structural particularities, led several authors to define a RINGO/Speedy box that is clearly distinct from the CBD, supporting their exclusion from the cyclin family.

The application of our criteria reinforces the existence of the canonical and the transcriptional cyclin subfamilies, designations that have been widely used in the literature. Moreover, our analysis allowed us to define, for the first time, the family of atypical cyclins. Although atypical cyclins have been mentioned by several groups [66, 81, 103, 108, 109], they were never object of a comprehensive analysis to establish whether they could actually be included in the cyclin family or what defined them as atypical. We believe that the name atypical is the one that more accurately reflects the particularities of these cyclins and has already been used by several groups [66, 81, 103, 108, 109]. Other names, such as “new cyclins” [110] or “orphan cyclins” [67, 111], are not recommended given that after all these years, they can hardly be considered new, whereas some previously orphan cyclins have now known CDK partners.

According to our analysis, atypical cyclins are characterized by three main aspects. One is the presence of the defining Lys–Glu pair in a context that is different from the double glutamic context observed in both canonical and transcriptional cyclins (Fig. 1). This distinctive context may explain the second aspect that characterizes atypical cyclins, which is the absence of interactors or the interaction with atypical CDKs (Fig. 2). The third aspect is the presence of a single CBD (Fig. 3). Although there are exceptions and, thus, none of these criteria is able to define atypical cyclins per se, when applied altogether, they reinforce the existence of a cyclins subfamily with distinctive features, supporting the choice for the name atypical.

Moreover, the interactor landscape suggests that atypical cyclins mostly interact with members of the subfamily of CDK5, which was considered the prototype of atypical CDKs [4]. Therefore, this update on cyclins nomenclature leads to a significant convergence with standing CDK nomenclature.

Whereas transcriptional cyclins clearly differ from other cyclins in the region that determines CDK binding (Fig. 1) or structural identity (Fig. 3), the line that separates canonical and atypical cyclins is much fainter. This observation is in line with the fact that canonical and atypical CDKs are also more closely related with each other than with transcriptional CDKs [4]. Given that atypical cyclins have not been thoroughly characterized, cyclin classifications are likely to evolve and cyclins that are now considered atypical may become canonical. Indeed, the similarities between atypical and canonical cyclins go beyond structural identity, as both have been implicated in the regulation of cell proliferation. Moreover, a closer look at this subfamily may unravel novel strategies to fight cancer. In this regard, our group has recently assigned an oncogenic role to CNTD2/CCNP in colon and lung cancers [67, 81]. Given that CCNP is overexpressed in several cancers and poorly expressed in tissues, it may represent the ideal target candidate. Further studies are now warranted to establish the physiological functions of atypical cyclins and understand their potential as therapeutic targets in cancer.

## References

[1] Evans T, Rosenthal ET, Youngblom J, Distel D, Hunt T (1983). Cyclin: a protein specified by maternal mRNA in sea urchin eggs that is destroyed at each cleavage division. Cell.

[2] Cao L, Chen F, Yang X, Xu W, Xie J, Yu L (2014). Phylogenetic analysis of CDK and cyclin proteins in premetazoan lineages. BMC Evol Biol.

[3] Lim S, Kaldis P (2013). Cdks, cyclins and CKIs: roles beyond cell cycle regulation. Development.

[4] Malumbres M (2014). Cyclin-dependent kinases. Genome Biol.

[5] Gibson TJ, Thompson JD, Blocker A, Kouzarides T (1994). Evidence for a protein domain superfamily shared by the cyclins, TFIIB and RB/p107. Nucleic Acids Res.

[6] Noble MEM, Endicott JA, Brown NR, Johnson LN (1997). The cyclin box fold: protein recognition in cell-cycle and transcriptional control. Trends Biochem Sci.

[7] Wood DJ, Endicott JA (2018). Structural insights into the functional diversity of the CDK—cyclin family. Open Biol.

[8] Kobayashi H, Stewart E, Poon R, Adamczewski JP, Gannon J, Hunt T (1992). Identification of the domains in cyclin A required for binding to, and activation of, p34cdc2 and p32cdk2 protein kinase subunits. Mol Biol Cell.

[9] Horne MC, Goolsby GL, Donaldson KL, Tran D, Neubauer M, Wahl AF (1996). Cyclin G1 and cyclin G2 comprise a new family of cyclins with contrasting tissue-specific and cell cycle-regulated expression. J Biol Chem.

[10] Ganesan V, Willis SD, Chang KT, Beluch S, Cooper KF, Strich R (2019). Cyclin C directly stimulates Drp1 GTP affinity to mediate stress-induced mitochondrial hyper-fission. Mol Biol Cell.

[11] Stover NA, Rice JD (2011). Distinct cyclin genes define each stage of ciliate conjugation. Cell Cycle.

[12] Al-shihabi A, Chawla SP, Hall FL, Gordon EM (2018). Exploiting oncogenic drivers along the CCNG1 pathway for cancer therapy and gene therapy. Mol Ther Oncolytics.

[13] Alvarez CA, Suvorova ES (2017). Checkpoints of apicomplexan cell division identified in Toxoplasma gondii. PLoS Pathog.

[14] D’Angiolella V, Donato V, Vijayakumar S, Saraf A, Florens L, Washburn MP, Dynlacht B, Pagano M (2010). SCF(Cyclin F) controls centrosome homeostasis and mitotic fidelity through CP110 degradation. Nature.

[15] McGrath DA, Fifield BA, Marceau AH, Tripathi S, Porter LA, Rubin SM (2017). Structural basis of divergent cyclin-dependent kinase activation by Spy1/RINGO proteins. EMBO J.

[16] Spurrier J, Shukla AK, McLinden K, Johnson K, Giniger E (2018). Altered expression of the Cdk5 activator-like protein, Cdk5α, causes neurodegeneration, in part by accelerating the rate of aging. Dis Model Mech.

[17] Vladar EK, Stratton MB, Saal ML, Salazar-De Simone G, Wang X, Wolgemuth D, Stearns T, Axelrod JD (2018). Cyclin-dependent kinase control of motile ciliogenesis. Elife.

[18] Lolli G (2010). Structural dissection of cyclin dependent kinases regulation and protein recognition properties. Cell Cycle.

[19] Hydbring P, Malumbres M, Sicinski P (2016). Non-canonical functions of cell cycle cyclins and cyclin-dependent kinases. Nat Rev Mol Cell Biol.

[20] Bregman DB, Pestell RG, Kidd VJ (2000). Cell cycle regulation and RNA polymerase II. Front Biosci.

[21] Badjatia N, Park SH, Ambrósio DL, Kirkham JK, Günzl A (2016). Cyclin-dependent kinase CRK9, required for spliced leader trans splicing of pre-mRNA in trypanosomes, functions in a complex with a new l-type cyclin and a kinetoplastid-specific protein. PLoS Pathog.

[22] Campsteijn C, Ovrebø JI, Karlsen BO, Thompson EM (2012). Expansion of cyclin D and CDK1 paralogs in Oikopleura dioica, a chordate employing diverse cell cycle variants. Mol Biol Evol.

[23] Ježek J, Smethurst DGJ, Stieg DC, Kiss ZAC, Hanley SE, Ganesan V, Chang KT, Cooper KF, Strich R (2019). Cyclin C: the story of a non-cycling cyclin. Biology (Basel).

[24] Mikolcevic P, Rainer J, Geley S (2012). Orphan kinases turn eccentric: a new class of cyclin Y-activated, membrane-targeted CDKs. Cell Cycle.

[25] Murray AW, Marks D (2001). Can sequencing shed light on cell cycling?. Nature.

[26] Neuman E, Ladha MH, Lin N, Upton TM, Miller SJ, DiRenzo J, Pestell RG, Hinds PW, Dowdy SF, Brown M, Ewen ME (1997). Cyclin D1 stimulation of estrogen receptor transcriptional activity independent of cdk4. Mol Cell Biol.

[27] Cooper KF, Khakhina S, Kim SK, Strich R (2014). Stress-induced nuclear-to-cytoplasmic translocation of Cyclin c promotes mitochondrial fission in yeast. Dev Cell.

[28] Nebreda AR (2006). CDK activation by non-cyclin proteins. Curr Opin Cell Biol.

[29] Malumbres M, Harlow E, Hunt T, Hunter T, Lahti JM, Manning G, Morgan DO, Tsai LH, Wolgemuth DJ (2009). Cyclin-dependent kinases: a family portrait. Nat Cell Biol.

[30] Shehata SN, Hunter RW, Ohta E, Peggie MW, Lou HJ, Sicheri F, Zeqiraj E, Turk BE, Sakamoto K (2012). Analysis of substrate specificity and cyclin Y binding of PCTAIRE-1 kinase. Cell Signal.

[31] Hinds PW, Dowdy SF, Eaton ENG, Arnold A, Weinberg RA (1994). Function of a human cyclin gene as an oncogene. Proc Natl Acad Sci USA.

[32] Jin J, Cardozo T, Lovering RC, Elledge SJ, Pagano M, Harper JW (2004). Systematic analysis and nomenclature of mammalian F-box proteins. Genes Dev..

[33] D’Angiolella V, Donato V, Forrester FM, Jeong YT, Pellacani C, Kudo Y, Saraf A, Florens L, Washburn MP, Pagano M (2012). Cyclin F-mediated degradation of ribonucleotide reductase M2 controls genome integrity and DNA repair. Cell.

[34] D’Angiolella V, Esencay M, Pagano M (2013). A cyclin without cyclin-dependent kinases: cyclin F controls genome stability through ubiquitin-mediated proteolysis. Trends Cell Biol.

[35] Augustine T, Chaudhary P, Gupta K, Islam S, Ghosh P, Santra MK, Mitra D (2017). Cyclin F/FBXO1 interacts with HIV-1 viral infectivity factor (Vif) and restricts progeny virion infectivity by ubiquitination and proteasomal degradation of vif protein through SCF cyclin F E3 ligase machinery. J Biol Chem.

[36] Edelheit S, Meiri N (2004). Cyclin S: a new member of the cyclin family plays a role in long-term memory. Eur J Neurosci.

[37] Li X, Wang X, Liu G, Li R, Yu L (2009). Identification and characterization of *cyclin X* which activates transcriptional activities of c-Myc. Mol Biol Rep.

[38] Ma Z, Wu Y, Jin J, Yan J, Kuang S, Zhou M, Zhang Y, Guo AY (2013). Phylogenetic analysis reveals the evolution and diversification of cyclins in eukaryotes. Mol Phylogenet Evol.

[39] Dong Q, Ph D, Kirley S, Rueda B, Ph D, Zhao C, Zukerberg L, Oliva E (2003). Loss of cables, a novel gene on chromosome 18q, in ovarian cancer. Mod Pahtol.

[40] Arnason T, Pino MS, Yilmaz O, Kirley SD, Rueda BR, Chung DC, Zukerberg LR (2013). Cables1 is a tumor suppressor gene that regulates intestinal tumor progression in Apc Min mice. Cancer Biol Ther.

[41] Shi Z, Park HR, Du Y, Li Z, Cheng K, Sun S, Li Z, Khuri FR (2015). Cables1 complex couples survival signaling to the cell death machinery. Cancer Res.

[42] Shi Z, Li Z, Li ZJ, Cheng K, Du Y, Fu HKF (2015). Cables1 controls p21/Cip1 protein stability by antagonizing proteasome subunit alpha type 3. Oncogene.

[43] Wang N, Guo L, Rueda BR, Tilly JL (2010). Cables1 protects p63 from proteasomal degradation to ensure deletion of cells after genotoxic stress. EMBO Rep.

[44] Huang J, Tan G, Li Y, Shi Z (2017). The emerging role of Cables1 in cancer and other diseases. Mol Pharmacol.

[45] Matsuoka M, Sudo H, Tsuji K, Sato H, Kurita M, Suzuki H, Nishimoto I, Ogata E (2003). ik3-2, a relative to ik3-1/Cables, is involved in both p53-mediated and p53-independent apoptotic pathways. Biochem Biophys Res Commun.

[46] Sato H, Nishimoto I, Matsuoka M (2002). ik3-2, a relative to ik3-1/cables, is associated with cdk3, cdk5, and c-abl. Biochim Biophys Acta.

[47] Okamoto K, Beach D (1994). Cyclin G is a transcriptional target of the p53 tumor suppressor protein. EMBO J.

[48] Zauberman A, Lupo A, Oren M (1995). Identification of p53 target genes through immune selection of genomic DNA: the cyclin G gene contains two distinct p53 binding sites. Oncogene.

[49] Okamoto K, Prives C (1999). A role of cyclin G in the process of apoptosis. Oncogene.

[50] Zhao L, Samuels T, Winckler S, Korgaonkar C, Tompkins V, Horne MC, Quelle DE (2003). Cyclin G1 has growth inhibitory activity linked to the ARF-Mdm2-p53 and pRb tumor suppressor pathways. Mol Cancer Res.

[51] Liu F, Gao X, Yu H, Yuan D, Zhang J, He Y, Yue L (2012). The role of progesterone and its receptor on cyclin G1 expression in endometrial carcinoma cells. Reprod Sci.

[52] Seo HR, Lee DH, Lee HJ, Baek M, Bae S, Soh JW, Lee SJ, Kim J, Lee YS (2006). Cyclin G1 overcomes radiation-induced G2 arrest and increases cell death through transcriptional activation of cyclin B1. Cell Death Differ.

[53] Okamoto K, Li H, Jensen MR, Zhang T, Taya Y, Thorgeirsson SS, Prives C (2002). Cyclin G recruits PP2A to dephosphorylate Mdm2. Mol Cell.

[54] Seo HR, Kim J, Bae S, Soh J-W, Lee Y-S (2008). Cdk5-mediated phosphorylation of c-Myc on Ser-62 is essential in transcriptional activation of cyclin B1 by cyclin G1. J Biol Chem.

[55] Bennin DA, Don ASA, Brake T, McKenzie JL, Rosenbaum H, Ortiz L, DePaoli-Roach AA, Horne MC (2002). Cyclin G2 associates with protein phosphatase 2A catalytic and regulatory B’ subunits in active complexes and induces nuclear aberrations and a G1/S phase cell cycle arrest. J Biol Chem.

[56] Arachchige Don AS, Dallapiazza RF, Bennin DA, Brake T, Cowan CE, Horne MC (2006). Cyclin G2 is a centrosome-associated nucleocytoplasmic shuttling protein that influences microtubule stability and induces a p53-dependent cell cycle arrest. Exp Cell Res.

[57] Nadeem U, Ye G, Salem M, Peng C (2014). MicroRNA-378a-5p targets Cyclin G2 to inhibit fusion and differentiation in BeWo Cells1. Biol Reprod.

[58] Yue L, Daikoku T, Hou X, Li M, Wang H, Nojima H, Dey SK, Das SK (2005). Cyclin G1 and cyclin G2 are expressed in the periimplantation mouse uterus in a cell-specific and progesterone-dependent manner: evidence for aberrant regulation with Hoxa-10 deficiency. Endocrinology.

[59] Bernaudo S, Salem M, Qi X, Zhou W, Zhang C, Yang W, Rosman D, Deng Z, Ye G, Yang BB, Vanderhyden B, Wu Z, Peng C (2016). Cyclin G2 inhibits epithelial-to-mesenchymal transition by disrupting Wnt/beta-catenin signaling. Oncogene.

[60] Hasegawa S, Eguchi H, Nagano H, Konno M, Tomimaru Y, Wada H, Hama N, Kawamoto K, Kobayashi S, Nishida N, Koseki J, Nishimura T, Gotoh N, Ohno S, Yabuta N, Nojima H, Mori M, Doki Y, Ishii H (2014). MicroRNA-1246 expression associated with CCNG2-mediated chemoresistance and stemness in pancreatic cancer. Br J Cancer.

[61] Choi M-G, Noh JH, An JY, Hong SK, Park SB, Baik YH, Kim KM, Sohn TS, Kim S (2009). Expression levels of cyclin G2, but not cyclin E, correlate with gastric cancer progression. J Surg Res.

[62] Cui DW, Cheng YJ, Jing SW, Sun GG (2014). Effect of cyclin G2 on proliferative ability of prostate cancer PC-3 cell. Tumour Biol.

[63] Cui DW, Sun GG, Cheng YJ (2014). Change in expression of cyclin G2 in kidney cancer cell and its significance. Tumour Biol.

[64] Sun GG, Hu WN, Cui DW, Zhang J (2014). Decreased expression of CCNG2 is significantly linked to the malignant transformation of gastric carcinoma. Tumour Biol.

[65] Griffin SV, Olivier JP, Pippin JW, Roberts JM, Shankland SJ (2006). Cyclin I protects podocytes from apoptosis. J Biol Chem.

[66] Brinkkoetter PT, Olivier P, Wu JS, Henderson S, Krofft RD, Pippin JW, Hockenbery D, Roberts JM, Shankland SJ (2009). Cyclin I activates Cdk5 and regulates expression of Bcl-2 and Bcl-XL in postmitotic mouse cells. J Clin Invest.

[67] Gasa L, Sanchez-Botet A, Quandt E, Hernández-Ortega S, Jiménez J, Carrasco-García MA, Simonetti S, Kron SJ, Ribeiro MP, Nadal E, Villanueva A, Clotet J (2017). A systematic analysis of orphan cyclins reveals CNTD2 as a new oncogenic driver in lung cancer. Sci Rep.

[68] Nagano T, Hashimoto T, Nakashima A, Hisanaga S, Kikkawa U, Kamada S (2013). Cyclin I is involved in the regulation of cell cycle progression. Cell Cycle.

[69] Zhang L, Xiao H, Zhou H, Santiago S, Lee JM, Garon EB, Yang J, Brinkmann O, Yan X, Akin D, Chia D, Elashoff D, Park NH, Wong DTW (2012). Development of transcriptomic biomarker signature in human saliva to detect lung cancer. Cell Mol Life Sci.

[70] Liu C, Zhai X, Zhao B, Wang Y, Xu Z (2017). Cyclin I-like (CCNI2) is a cyclin-dependent kinase 5 (CDK5) activator and is involved in cell cycle regulation. Sci Rep.

[71] Ohshima T, Ward JM, Huht C, Longenecker G, Pant HC, Bradyt R, Martin LJ, Kulkarni AB (1996). Targeted disruption of the cyclin-dependent kinase 5 gene results in abnormal corticogenesis, neuronal pathology and perinatal death. Proc Natl Acad Sci USA.

[72] Pozo K, Castro-rivera E, Tan C, Plattner F, Schwach G, Siegl V, Meyer D, Guo A, Gundara J, Mettlach G, Richer E, Guevara JA, Ning L, Gupta A, Hao G, Tsai LH, Sun X, Antich P, Sidhu S, Robinson BG, Chen H, Nwariaku FE, Pfragner R, Richardson JA, Bibb JA (2013). The Role of Cdk5 in Neuroendocrine Thyroid Cancer. Cancer Cell.

[73] Wallmeier J, Al-Mutairi DA, Chen C-T, Loges NT, Pennekamp P, Menchen T, Ma L, Shamseldin HE, Olbrich H, Dougherty GW, Werner C, Alsabah BH, Köhler G, Jaspers M, Boon M, Griese M, Schmitt-Grohé S, Zimmermann T, Koerner-Rettberg C, Horak E, Kintner C, Alkuraya FS, Omran H (2014). Mutations in CCNO result in congenital mucociliary clearance disorder with reduced generation of multiple motile cilia. Nat Genet.

[74] Funk MC, Bera AN, Menchen T, Kuales G, Thriene K, Lienkamp SS, Dengjel J, Omran H, Frank M, Arnold SJ (2015). Cyclin O (Ccno) functions during deuterosome- mediated centriole amplification of multiciliated cells. EMBO J.

[75] Amirav I, Wallmeier J, Loges NT, Menchen T, Pennekamp P, Mussaffi H, Abitbul R, Avital A, Bentur L, Dougherty GW, Nael E, Lavie M, Olbrich H, Werner C, Kintner C, Omran H, Israeli PCD Consortium Investigators (2016). Systematic analysis of CCNO variants in a defined population: implications for clinical phenotype and differential diagnosis. Hum Mutat.

[76] Casey JP, McGettigan PA, Healy F, Hogg C, Reynolds A, Kennedy BN, Ennis S, Slattery D, Lynch SA (2015). Unexpected genetic heterogeneity for primary ciliary dyskinesia in the Irish Traveller population. Eur J Hum.

[77] Guo T, Tan Z-P, Chen H-M, Zheng D, Liu L, Huang XG, Chen P, Luo H, Yang YF (2017). An effective combination of whole-exome sequencing and runs of homozygosity for the diagnosis of primary ciliary dyskinesia in consanguineous families. Sci Rep.

[78] Villa M, Crotta S, Dingwell KS, Hirst EMA, Gialitakis M, Ahlfors H, Smith JC, Stockinger B, Wack A (2016). The aryl hydrocarbon receptor controls cyclin O to promote epithelial multiciliogenesis. Nat Commun.

[79] Núnez-Ollé M, Jung C, Terré B, Balsiger NA, Plata C, Roset R, Pardo-Pastor C, Garrido M, Rojas S, Alameda F, Lloreta J, Martín-Caballero J, Flores JM, Stracker TH, Valverde MA, Muñoz FJ, Gil-Gómez G (2017). Constitutive Cyclin O deficiency results in penetrant hydrocephalus, impaired growth and infertility. Oncotarget.

[80] Roig MB, Roset R, Ortet L, Balsiger NA, Anfosso A, Cabellos L, Garrido M, Alameda F, Brady HJ, Gil-Gómez G (2009). Identification of a novel cyclin required for the intrinsic apoptosis pathway in lymphoid cells. Cell Death Differ.

[81] Sánchez-Botet A, Gasa L, Quandt E, Hernández-Ortega S, Jiménez J, Mezquita P, Carrasco-García MÀ, Kron SJ, Vidal A, Villanueva A, Ribeiro MPC, Clotet J (2018). The atypical cyclin CNTD2 promotes colon cancer cell proliferation and migration. Sci Rep.

[82] Murray J, Todd KV, Bakre A, Orr-Burks N, Jones L, Wu W, Tripp RA (2017). A universal mammalian vaccine cell line substrate. PLoS One.

[83] Zhang R, Strong MJ, Baddoo M, Lin Z, Wang Y-P, Flemington EK, Liu YZ (2017). Interaction of Epstein-Barr virus genes with human gastric carcinoma transcriptome. Oncotarget.

[84] Mikolcevic P, Sigl R, Rauch V, Hess MW, Pfaller K, Barisic M, Pelliniemi LJ, Boesl M, Geley S (2012). Cyclin-dependent kinase 16/PCTAIRE kinase 1 is activated by cyclin Y and is essential for spermatogenesis. Mol Cell Biol.

[85] Hernández-Ortega S, Sánchez-Botet A, Quandt E, Masip N, Gasa L, Verde G, Jiménez J, Levin RS, Rutaganira FU, Burlingame AL, Wolfgeher D, Ribeiro MPC, Kron SJ, Shokat KM, Clotet J (2019). Phosphoregulation of the oncogenic Protein Regulator of Cytokinesis 1 (PRC1) by the atypical CDK16/CCNY complex. Exp Mol Med.

[86] Sun T, Co NN, Wong N (2014). PFTK1 interacts with cyclin y to activate non-canonical Wnt signaling in hepatocellular carcinoma. Biochem Biophys Res Commun.

[87] Zeng L, Cai C, Li S, Wang W, Li Y, Chen J, Zhu X, Zeng YA (2016). Essential roles of Cyclin Y-like 1 and Cyclin Y in dividing Wnt-responsive mammary stem/progenitor cells. PLoS Genet.

[88] Cho E, Kim DH, Hur YN, Whitcomb DJ, Regan P, Hong JH, Kim H, Ho Suh Y, Cho K, Park M (2015). Cyclin Y inhibits plasticity-induced AMPA receptor exocytosis and LTP. Sci Rep.

[89] Joe IS, Kim JH, Kim H, Hong JH, Kim M, Park M (2017). Cyclin Y-mediated transcript profiling reveals several important functional pathways regulated by Cyclin Y in hippocampal neurons. PLoS One.

[90] Zi Z, Zhang Z, Li Q, An W, Zeng L, Gao D, Yang Y, Zhu X, Zeng R, Shum WW, Wu J (2015). CCNYL1, but Not CCNY, cooperates with CDK16 to regulate spermatogenesis in mouse. PLoS Genet.

[91] Koch S, Acebron SP, Herbst J, Hatiboglu G, Niehrs C (2015). Post-transcriptional Wnt signaling governs epididymal sperm maturation. Cell.

[92] Holloway JK, Sun X, Yokoo R, Villeneuve AM, Cohen PE (2014). Mammalian CNTD1 is critical for meiotic crossover maturation and deselection of excess precrossover sites. J Cell Biol.

[93] Shibata H, Yamamoto K, Sun Z, Oka A, Inoko H, Arinami T, Inada T, Ujike H, Itokawa M, Tochigi M, Watanabe Y, Someya T, Kunugi H, Suzuki T, Iwata N, Ozaki N, Fukumaki Y (2013). Genome-wide association study of schizophrenia using microsatellite markers in the Japanese population. Psychiatr Genet.

[94] Wang Z, Liu W, Chong C, Yang X, Luo Y, Bailin Z (2019). Low mutation and neoantigen burden and fewer effector tumor infiltrating lymphocytes correlate with breast cancer metastasization to lymph nodes. Sci Rep.

[95] Feliciano A, Castellvi J, Artero-Castro A, Leal JA, Romagosa C, Hernández-Losa J, Peg V, Fabra A, Vidal F, Kondoh H, Ramón Y, Cajal S, Lleonart ME (2013). miR-125b Acts as a Tumor Suppressor in Breast Tumorigenesis via Its Novel Direct Targets ENPEP, CK2-α, CCNJ, and MEGF9. PLoS One.

[96] Sun X, Du P, Yuan W, Du Z, Yu M, Yu X, Hu T (2015). Long non-coding RNA HOTAIR regulates cyclin J via inhibition of microRNA-205 expression in bladder cancer. Cell Death Dis.

[97] Ting HJ, Messing J, Yasmin-Karim S, Lee YF (2013). Identification of microRNA-98 as a therapeutic target inhibiting prostate cancer growth and a biomarker induced by vitamin D. J Biol Chem.

[98] Venturutti L, Cordo Russo RI, Rivas MA, Mercogliano MF, Izzo F, Oakley RH, Pereyra MG, De Martino M, Proietti CJ, Yankilevich P, Roa JC, Guzmán P, Cortese E, Allemand DH, Huang TH, Charreau EH, Cidlowski JA, Schillaci R, Elizalde PV (2016). MiR-16 mediates trastuzumab and lapatinib response in ErbB-2- positive breast and gastric cancer via its novel targets CCNJ and FUBP1. Oncogene.

[99] Liu L, Michowski W, Inuzuka H, Shimizu K, Nihira NT, Chick JM, Li N, Geng Y, Meng AY, Ordureau A, Kołodziejczyk A, Ligon KL, Bronson RT, Polyak K, Harper JW, Gygi SP, Wei W, Sicinski P (2017). G1 cyclins link proliferation, pluripotency and differentiation of embryonic stem cells. Nat Cell Biol.

[100] Davidson G, Shen J, Huang YL, Su Y, Karaulanov E, Bartscherer K, Hassler C, Stannek P, Boutros M, Niehrs C (2009). Cell cycle control of wnt receptor activation. Dev Cell.

[101] Liu H, Shi H, Fan Q, Sun X (2016). Cyclin Y regulates the proliferation, migration, and invasion of ovarian cancer cells via Wnt signaling pathway. Tumor Biol.

[102] Fornari F, Gramantieri L, Giovannini C, Veronese A, Ferracin M, Sabbioni S, Calin GA, Grazi GL, Croce CM, Tavolari S, Chieco P, Negrini M, Bolondi L (2009). MiR-122/cyclin G1 interaction modulates p53 activity and affects doxorubicin sensitivity of human hepatocarcinoma cells. Cancer Res.

[103] Russell P, Hennessy BT, Li J, Carey MS, Bast RC, Freeman T, Venkitaraman AR (2012). Cyclin G1 regulates the outcome of taxane-induced mitotic checkpoint arrest. Oncogene.

[104] Dinarina A, Perez LH, Davila A, Schwab M, Hunt T, Nebreda AR (2005). Characterization of a new family of cyclin-dependent kinase activators. Biochem J.

[105] Cheng A, Xiong W, Ferrell JE, Solomon MJ (2005). Identification and comparative analysis of multiple mammalian speedy/ringo proteins. Cell Cycle.

[106] Karaiskou A, Perez LH, Ferby I, Ozon R, Jessus C, Nebreda AR (2001). Differential regulation of Cdc2 and Cdk2 by RINGO and cyclins. J Biol Chem.

[107] Cheng A, Gerry S, Kaldis P, Solomon MJ (2005). Biochemical characterization of Cdk2-Speedy/Ringo A2. BMC Biochem.

[108] Nagel AC, Fischer P, Szawinski J, La Rosa MK, Preiss A (2012). Cyclin G is involved in meiotic recombination repair in Drosophila melanogaster. J Cell Sci.

[109] Dhara A, de Paula Baptista R, Kissinger JC, Snow EC, Sinai AP (2017). Ablation of an ovarian tumor family deubiquitinase exposes the underlying regulation governing the plasticity of cell cycle progression in toxoplasma gondii. MBio.

[110] Nakamura T, Sanokawa R, Sasaki YF, Ayusawa D, Oishi M, Mori N (1995). Cyclin I: a new cyclin encoded by a gene isolated from human brain. Exp Cell Res.

[111] Malumbres M, Barbacid M (2005). Mammalian cyclin-dependent kinases. Trends Biochem Sci.

[112] Crooks G, Hon G, Chandonia J, Brenner S (2004). WebLogo: a sequence logo generator. Genome Res.

[113] Finn RD, Bateman A, Clements J, Coggill P, Eberhardt RY, Eddy SR, Heger A, Hetherington K, Holm L, Mistry J, Sonnhammer EL, Tate J, Punta M (2014). Pfam: the protein families database. Nucleic Acids Res.

[114] Szklarczyk D, Gable AL, Lyon D, Junge A, Wyder S, Huerta-Cepas J, Simonovic M, Doncheva NT, Morris JH, Bork P, Jensen LJ, Mering CV (2019). STRING v11: protein–protein association networks with increased coverage, supporting functional discovery in genome-wide experimental datasets. Nucleic Acids Res.

[115] Varjosalo M, Sacco R, Stukalov A, van Drogen A, Planyavsky M, Hauri S, Aebersold R, Bennett KL, Colinge J, Gstaiger M, Superti-Furga G (2013). Interlaboratory reproducibility of large-scale human protein-complex analysis by standardized AP-MS. Nat Methods.

[116] Guen VJ, Gamble C, Flajolet M, Unger S, Thollet A, Ferandin Y, Superti-Furga A, Cohen PA, Meijer L, Colas P (2013). CDK10/cyclin M is a protein kinase that controls ETS2 degradation and is deficient in STAR syndrome. Proc Natl Acad Sci USA.

[117] Bates S, Rowan S, Vousden KH (1996). Characterisation of human cyclin G1 and G2: DNA damage inducible genes. Oncogene.

[118] Reimer CL, Borras AM, Kurdistani SK, Garreau JR, Chung M, Aaronson SA, Lee SW (1999). Altered regulation of cyclin G in human breast cancer and its specific localization at replication foci in response to DNA damage in p53+/+ cells. J Biol Chem.

[119] Kimura SH, Ikawa M, Ito A, Okabe M, Nojima H (2001). Cyclin G1 is involved in G2/M arrest in response to DNA damage and in growth control after damage recovery. Oncogene.

[120] Zimmermann M, Arachchige-Don AS, Donaldson MS, Dallapiazza RF, Cowan CE, Horne MC (2012). Elevated cyclin G2 expression intersects with DNA damage checkpoint signaling and is required for a potent G2/M checkpoint arrest response to doxorubicin. J Biol Chem.

[121] Naito Y, Yabuta N, Sato J, Ohno S, Sakata M, Kasama T, Ikawa M, Nojima H (2013). Recruitment of cyclin G2 to promyelocytic leukemia nuclear bodies promotes dephosphorylation of γH2AX following treatment with ionizing radiation. Cell Cycle.

[122] Park M, Watanabe S, Poon VY, Ou CY, Jorgensen EM, Shen K (2011). CYY-1/cyclin Y and CDK-5 differentially regulate synapse elimination and formation for rewiring neural circuits. Neuron.

